# Information following a Diagnosis of Congenital Heart Defect: Experiences among Parents to Prenatally Diagnosed Children

**DOI:** 10.1371/journal.pone.0117995

**Published:** 2015-02-18

**Authors:** Tommy Carlsson, Gunnar Bergman, Ulla Melander Marttala, Barbro Wadensten, Elisabet Mattsson

**Affiliations:** 1 Department of Public Health and Caring Sciences, Uppsala University, Uppsala, Sweden; 2 Department of Women’s and Children’s Health, Karolinska Institutet, Stockholm, Sweden; 3 Department of Scandinavian Languages, Uppsala University, Uppsala, Sweden; 4 Department of Women’s and Children’s Health, Uppsala University, Uppsala, Sweden

## Abstract

**Background:**

Prenatal screening of pregnant women in Sweden has improved the detection of major congenital heart defects (CHD). The aim was to explore parental experiences and need for information following a prenatal diagnosis of CHD.

**Methods:**

Semi-structured interviews conducted with six fathers and five mothers to seven prenatally diagnosed children. Data were analyzed through content analysis.

**Results:**

Three themes and 9 categories emerged. Theme 1, Grasping the facts today while reflecting on the future, containing five categories: Difficulties sorting out information when in emotional chaos; Respectful information regarding termination of pregnancy; Early information is crucial; Understanding the facts regarding the anomaly; Preparing for the future. Theme 2, Personal contact with medical specialists who give honest and trustworthy information is valued, containing two categories: Trust in information received from medical specialists and Truth and honesty is valued. Theme 3, An overwhelming amount of information on the Internet, containing two categories: Difficulties in finding relevant information and Easy to focus on cases with a poor outcome when searching the Internet.

**Conclusion:**

Early and honest information in line with individual preferences is crucial to support the decisional process regarding whether to continue or terminate the pregnancy. The use of illustrations is recommended, as a complement to oral information, as it increases comprehension and satisfaction with obtained information. Furthermore, the overwhelming amount of information on the Internet calls for compilation of easily accessible and reliable information sources via the Internet.

## Background

In Sweden, all pregnant women are offered a routine ultrasound screening at approximately 18 weeks of gestation, to which 97% consent [1]. The main purpose of the screening is to estimate gestational age, detect multiple pregnancies, and screen for fetal anomalies [2]. Most pregnant women have optimistic expectations about the screening and rarely feel worried about the potential results of the ultrasound. They may thus, be unprepared for unexpected adverse findings [3–5].

Ultrasound screening has improved the detection rate of major congenital heart defects (CHD). In cases where corrective heart surgery could be offered, the majority of women have been shown to choose to continue the pregnancy, with termination of the pregnancy predominantly being due to the most severe cases of structural heart defects (e.g. univentricular hearts) and in cases with associated extracardiac or chromosomal abnormalities [6]. The time between diagnosis, at approximately 18–20 gestational weeks, and having to make a decision regarding the future of the pregnancy is limited. According to current Swedish legislation the mother has the right to decide on termination of the pregnancy prior to a gestational age of 18 weeks and 0 days. However, the mother needs approval from the National Board of Health and Welfare for induced termination of the pregnancy from a gestational age of 18 weeks and 0 days. In accordance with clinical practice, approval is not given after a gestational age of 22 weeks and 0 days.

The process towards an informed decision on the future of a pregnancy involves various difficulties for the woman including ethical considerations [7], psychological distress [8], and trying to understand and value complex medical information [9]. Partners of pregnant women with detected abnormalities in the fetus report a need for information and desire joint decisions regarding the pregnancy [10]. However, descriptions of parents’ experiences of information and need for information following the diagnosis are limited, and few studies give voice to fathers’ experiences [11]. Thus, the aim of this study was to explore parental experiences and the need for information following a prenatal diagnosis of congenital heart.

## Methods

The study had a qualitative design, the goal was thus to gain insight into a certain phenomenon [12], i.e. experiences and need for information following a prenatal diagnosis of CHD.

### Ethics Statement

Ethical approval to conduct the study was obtained from the Regional Ethical Review Board in Uppsala, Sweden. Written and oral consent was collected before enrolment.

### Context

The study was performed at a tertiary referral centre for fetal cardiology and fetal medicine at Uppsala University Hospital, Sweden, and was part of a larger project aimed at developing an information intervention for expectant parents following a prenatal diagnosis of CHD.

Consultative service was provided by pediatric cardiologists specialized in fetal cardiology. Based on the findings and possible precision of the examination, information on a broad variety of topics including the diagnosis with associated consequences, possible cause, natural course, possible surgical treatment, results and prognosis was provided orally and through illustrations. The risk of associated malformations and chromosomal abnormalities was highlighted and as a routine, additional fetal medicine investigations and chromosomal testing were offered through close co-operation with the fetal medicine unit. After the initial consultation, fetal cardiology follow-up was offered every 4–6 weeks in addition to the fetal medicine program, to monitor the progression of the CHD, prepare the couple and optimize the planning of the perinatal management, including decisions about intrauterine referral to a pediatric heart-surgery centre when stabilization and surgery were expected during the first postnatal week.

### Participants

**Fig 1:**
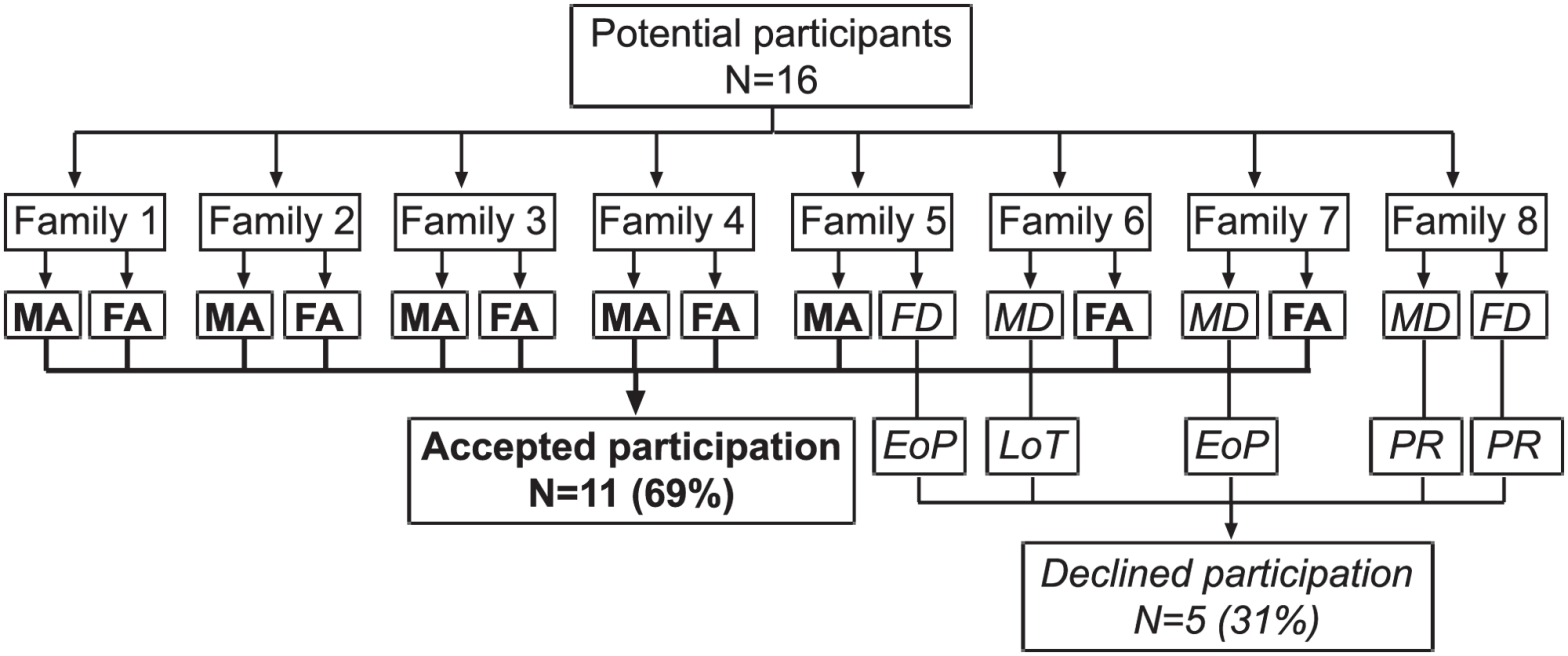
Potential participants and study participants. EoP = The parent felt that one participant from the family was enough, FA = Father accepted, FD = Father declined, LoT = Lack of time on part of the parent, MA = Mother accepted, MD = Mother declined, PR = Personal reasons.

The sample consists of Swedish-speaking parents with a child prenatally diagnosed at gestational week 18–20 with a major congenital heart defect, defined as a condition in need of surgical treatment during the first year of life. Purposive sampling [13] was used, and thus parents with different cultural and socio-demographic backgrounds were chosen in order to achieve variations of the phenomenon under study. The second and last authors were responsible for recruitment of participants from their network. See Fig. 1 for a presentation of potential participants, study participants, and reasons for attrition.

To gain insight into the perspectives and stories [12] of the parents, semi-structured interviews [14] were conducted with 6 fathers and 5 mothers to 7 prenatally diagnosed children, Table 1. The prenatal and confirmed postnatal diagnoses of included cases were atrioventricular septal defect (N = 2), coarctation of the aorta, Fallot’s anomaly (N = 2), pulmonary atresia with ventricular septal defect and major aortopulmonary collateral arteries and finally a case of ventricular septal defect. No cases of associated extracardial or chromosomal abnormalities were included.

**Table 1:** Characteristics of study participants.

Category	Subcategory	No. (%)
Parent	Fathers	6 (55%)
	Mothers	5 (45%)
Origin of parent	Foreign	4 (36%)
	Swedish	7 (64%)
Highest education of parent	Junior high school	1 (9%)
	Senior high school	4 (36%)
	University/College	6 (55%)
Age of parent (years)	20–29	2 (18%)
	30–39	7 (64%)
	>39	2 (18%)
Child’s surgical interventions	None (Awaiting intervention)	2 (18%)
	One	6 (55%)
	Two	3 (27%)
Age of child (years)	<1	4 (36%)
	1–2	6 (55%)
	>2	1 (9%)

### Data Collection

The potential participants were given written and oral information about the study and parents who agreed to participate and completed a written informed consent were contacted by the first author and asked to choose a suitable time and location for the interview. The interviews were conducted between April and June 2013, five individual interviews (two with mothers, three with fathers) and three interviews with both parents together. Three interviews were conducted at the hospital and five interviews at the home of the parents. The interviews lasted 29 to 60 minutes. With the permission of the participants, the interviews were audio recorded and transcribed verbatim after the interview. However, one participant did not consent to audio recording. The interviewer (TC) made handwritten notes during the interview instead.

The first author, who is a registered nurse and a trained interviewer, conducted all interviews. The parents met the interviewer in his role as a researcher and had no previous contact with him before or after the interview. The interviewer kept a reflective journal during the course of the study in order to elucidate possible preconceptions.

An interview guide [12] was developed by the authors in order to stay relevant to the aim, Table 2. All participants were asked the same questions in the same order. At the beginning of each interview the parents were asked structured questions about their age, educational level and the child’s age, diagnosis and number of surgical interventions. Following this, four open-ended questions were asked:”Could you tell me what it was like for you to be told about the heart defect?”, “Could you describe what information you experienced you needed when the diagnosis was made?”, “Could you describe how you experienced the oral and written information you received from the hospital staff in connection with the diagnosis?”, and “Could you describe how you looked for information by yourself?”. The interviewer was supportive and asked follow-up questions in order to help the parents to elucidate their answers. Interviews were carried out until data saturation was reached, i.e. when further data collection no longer deepened or challenged findings from previous interviews [13].

**Table 2:** Interview guide.

1. Could you tell me what it was like for you to be told about the heart defect?
2. Could you describe what information you experienced you needed when the diagnosis was made?
i. Are there any subjects you experience it is particularly important to be given information about when the diagnosis is made?
ii. In what way would you have preferred to have been given information?
3. Could you describe how you experienced the oral and written information you received from the hospital staff in connection with the diagnosis?
i. Who gave the information?
ii. Was the information easy or difficult to understand?
iii. Were you given any written information?
iv. Was any information particularly useful?
v. Did you feel that some information was missing?
vi. Was any information superfluous?
4. Could you describe how you looked for information by yourself?
i. What methods did you use to look for information?
ii. How did you experience the information you found on your own?

### Data Analysis

The transcribed interviews underwent qualitative content analysis [15], a method that aims at systematically describing differences and similarities in any form of communication, such as an interview transcript. The texts from the interviews were read several times. Meaning units, each one representing a single unit of content, were identified, condensed, and assigned a descriptive code. The first, fourth and the last author structured the codes into categories, which are collections of different codes that have a common subject, and formed themes from the categories. Themes are the underlying meaning of the categories, and represent the interpretation of the structure of the data, i.e. the latent content. This analysis moved back and forth dialectically. Discussions concerning codes, categories, and themes were held during the analysis process until consensus was reached. This resulted in three themes with 9 categories.

## Results

Three different themes emerged: “Grasping the facts today while reflecting on the future”, “Personal contact with medical specialists who give honest and trustworthy information is valued”, and “An overwhelming amount of information on the Internet”.

### Theme 1: Grasping the Facts Today While Reflecting on the Future

The theme consists of five categories: “Difficulties sorting out information when in emotional chaos”, “Respectful information regarding termination of pregnancy”, “Early information is crucial”, “Understanding the facts regarding the anomaly”, and “Preparing for the future”.

**Difficulties Sorting out Information When in Emotional Chaos**. The situation at the time of the diagnosis was described as emotionally tough, chaotic, and the worst day of the person’s life. The acute stress reaction made it even more difficult to comprehend and understand all the information given by the caregivers. The first weeks after the diagnosis were experienced as disordered, and the decision to either continue or terminate the pregnancy was very difficult. While some wanted to gain as much information as possible during the first consultation with the fetal cardiologist, others wanted gradual information, distributed over several occasions. It was easy to forget what was said during the consultations and many questions arose in the aftermath. Repeated information was thus considered of great importance.

Parents with Swedish as a second language mentioned that the caregivers used unfamiliar words. One parent described that she and her partner perceived the given information differently, which led to confusion:

*“Your reactions and feelings have such a big impact on the information you take in. You see, we’ve noticed when we get together, me and my husband, that we have understood things differently. So afterwards we’ve kind of talked about… Did he say that? No, he didn’t say that… Yes, but I thought he said that…. sort of.. So you notice that even though you’ve heard the same thing, you haven’t really done so at all”*

(Mother 2)

**Respectful Information Regarding Termination of Pregnancy**. None of the parents described dissatisfaction with the information about termination of pregnancy. A portion of the parents did not however feel a need for any information about termination of pregnancy, since they would not consider it for religious reasons. The remaining parents valued information about a possible late abortion, and felt that physicians should be neutral when discussing this. Parents appreciated when healthcare providers confirmed that it is normal to have ambivalent feelings whether to continue or terminate the pregnancy:

*“So you find out actually that what you’re thinking is normal … I think that is quite an important thing to learn. That … it’s normal to think about whether to terminate or not, it’s …”*

(Father 2)

**Early Information is Crucial**. The parents wanted to know promptly what was wrong. To be able to ask questions about the anomaly was crucial to make a decision as to whether continue or terminate the pregnancy. One parent felt that she had not understood the time limit for a late abortion:

*“First they do a quick analysis of some kind and then they do … the whole … I don’t really know about that but … we received it later. And I had thought that after that information, we could decide whether to keep it or not. But … then the doctor I talked to said that basically it was too late…”*

(Mother 1)

**Understanding the Facts Regarding the Anomaly**. Detailed information about the specific CHD and normal functions of the heart facilitated understanding. Drawings of the CHD combined with short written information helped the parents to remember what had been said during the consultation and to get a picture of the anomaly, as well as to understand difficult words, especially among those with Swedish as a second language.

*“Because you truly don’t really know how the organ works in the body. So he … Showed exactly how the blood flow was in and out and how it was oxygenated, so then I understood how the heart was constructed and then I also understood what it all meant, how slight or severe the heart defect was.”*

(Father 6)

The drawings were also an aid when explaining the condition to others. Information about associated chromosomal anomalies was important, and a significant factor when deliberating about termination of the pregnancy. Furthermore, parents mentioned that they questioned if the CHD was caused by the parents and pointed out the importance of bringing up this subject with the physician.

**Preparing for the Future**. The parents had many thoughts about the future: about life and death, and worries about future treatments for the child. They also pondered about the impact on the family, as well as future quality of life for the child:

*“Of course you worry about how things will turn out. When the child is bigger. Uhh, it can be fixed … so that he’ll be alright, but what quality of life will it be after that. That… that was what we talked about most…”*

(Father 3)

Information about the birth, potential future symptoms, and future surgeries were important since the parents later on felt more prepared to deal with the situation. The parents mentioned that it was helpful to get information about how stressful the time around the surgeries and the frequent hospital visits can be, especially when siblings are involved.

The parents described that they wanted to relate to people in similar circumstances previously, as this would help them deal with the tough emotional situation. It was considered important to hear about cases that ended well, but also about the cases that did not.

### Theme 2: Personal Contact With Medical Specialists Who Give Honest and Trustworthy Information is Valued

The theme consists of two categories: “Trust in information received from medical specialists”, and “Truth and honesty is valued”.

**Trust in Information Received From Medical Specialists**. Personal contact with caregivers was considered absolutely the best way to receive information, since the information came directly from a confirmed and trusted source i.e. from specialists. The parents mentioned the importance of continuity of care as they believed it facilitated understanding and reduced anxiety. Overall, the parents were satisfied by the information given by the caregivers.

*“The best thing is when you can talk to the doctor. It’s more … I trust a doctor more than what … And then you can ask questions which maybe aren’t … aren’t … Yes … If you sit and read something, then questions can pop up in your mind.”*

(Mother 1)

**Truth and Honesty is Valued**. It was emphasized that caregivers should deliver honest information and not withhold anything. One parent expressed a worry that the risks and complications in association with surgery might not be fully described:

*“Good lord, such a little heart, and there’s not so much … margin for error … It’s still a complicated operation. And … I don’t know … but … perhaps they paint a bit of a rosy picture…we can do it and get a good result, but there’s still really a lot of risks involved … when you do an operation like that. Because it’s not just to go in and do a heart operation.”*

(Mother 1)

### Theme 3: An Overwhelming Amount of Information on the Internet

The theme consists of two categories: “Difficult to find relevant information”, and “Easy to focus on cases with a poor outcome when searching the Internet”.

**Difficult to Find Relevant Information**. A portion of the parents frequently used the Internet to find information especially at the time of diagnosis, while others did not use it at all. The parents who used the Web considered it a good source of information, if they found relevant sources. The parents who avoided the Internet did so because of initial difficulties understanding the information found and problems finding information relevant to their specific situation. It was emphasized that the parents wanted information that was easily understood, and that the websites visited were difficult to understand due to foreign languages and/or medical terminology. The parents felt that they could not trust the information found via the Internet. Searching the Web for information resulted in more worry. Relying exclusively on the information from the caregivers was a way to cope with this situation.

*“And how much truth is there in it … And then I kind of don’t want to either… I don’t want to read a load of stuff that makes me more worried than I need to be. And maybe it goes a bit against what I said about wanting to know as much as possible, but … It feels much better to talk to the doctor or hospital staff who know our specific case.”*

(Mother 2)

The parents mentioned that it would be positive if the caregivers could recommend written material, websites, or Internet-based social networks. Further, they thought that a website provided by the caregivers would be helpful, since this would present an opportunity to go home and repeat the information received during the consultation.

**Easy to Focus on Cases With a Poor Outcome When Searching the Internet**. It was easy to focus on and/or difficult to avoid reading on the Web about cases with a poor outcome. This led to fear about the future of one’s own child. One parent described being distressed when reading in a blog about a child that died:

*“Sure, in some blog the child actually died … It was pretty terrible. But … I remember when I read it. Quite a few thoughts passed through my mind as a matter of fact.”*

(Father 2)

## Discussion

In the present study we have demonstrated that the complex combination of parents’ acute stress reaction, obstacles to comprehension, the decisional process regarding the future of the pregnancy, and an overwhelming amount of information on the Internet summarize issues when providing information following a prenatal diagnosis of congenital heart disease.

Our study, together with other studies [3,16], underscores the importance of early and honest information so that the pregnant woman can make an informed decision regarding whether to continue or terminate the pregnancy. However, health professionals need to consider that expectant parents can be overwhelmed by the sheer amount of information available. The Internet is a known source of information and insight among parents to children with CHD [17], especially in connection with the time of diagnosis [18]. However, difficulties finding relevant and specific information have been acknowledged [19,20]. Parents in our study desired recommendations from health-care providers for relevant and adequate informational sources on the Internet. They also expressed a need for social support i.e. they wanted to be put in touch with parents in the same situation.

Obstacles to understanding medical terminology may be due to parents’ emotional distress [21] and their efforts to comprehend have been described as similar to learning a new language [22]. However, health literacy skills, i.e. the degree to which individuals have the capacity to obtain, process, and understand health information [23] may also affect the information process. Low health literacy has been found to be a primary factor behind health disparities [24,25]. The concept is therefore of increasing concern to health professionals.

The parents’ satisfaction with information received in our study may partly be explained by the drawings of the specific CHD combined with short written supplemental information provided by the physicians. Illustrations as a complement to oral information can increase comprehension and are especially helpful among those with low health literacy [26]. Furthermore, supplemental written information helps to recall information [3], and has the potential to increase satisfaction and knowledge [27].

Persons with Swedish as a second language experienced difficulties in understanding the detected fetal heart disease. Not being able to fully master the language has been equated to a silent and hidden social disability, often associated with shame [28]. Consequently, persons with these difficulties need extra support as they may avoid asking the necessary questions to understand the provided information, with misunderstandings and confusion as a result. This illustrates the importance of interpreter services.

### Methodological Reflections

The credibility [15] of this study, i.e. how well data and processes of analysis addressed the intended focus, was achieved through purposive sampling with maximum variation [12]. Furthermore, data collection was carried out until saturation was achieved, i.e. no more categories emerged in the analysis [12]. In order to minimise the risk of only one perspective determining how data were categorised, two persons, TC and EM, both experienced in content analysis, one with and one without experience of working within the field of paediatric cardiology, took part in the data analysis. Finally, to ensure that the categories were clearly distinguished from each other and were given appropriate names and descriptions, i.e. reflecting the content of the meaning units, an additional assessor, BW, participated in this part of the analysis. Through these approaches a multifaceted picture of parental experiences and need for information following a prenatal diagnosis of CHD was attained.

The parents were able to choose whether to be interviewed together or individually. It is possible that new findings would have emerged if all parents had been interviewed in the same manner. On the other hand, the combination of these two approaches gave us the opportunity to obtain both a broad understanding of the information process following a prenatal diagnosis of CHD and deeper genuine accounts of some parents’ own experiences.

The literature related to parents’ experiences of information following a prenatal diagnosis of CHD has primarily focused on mothers [11]. However, in this study we also take into account fathers’ experiences and need for information. The findings indicate that fathers’ and mothers’ descriptions of the information process were concordant. However, as there is a shortage of studies involving fathers from different educational, ethnic, and social backgrounds [29], prospective studies are needed to further elucidate expectant fathers’ experiences and need for information during antenatal care.

The sample included parents to children with a major CHD who had undergone corrective biventricular repair, as well as palliative heart surgery, and parents who were waiting for their child’s surgery. However, no parent to a child with associated chromosomal abnormalities was included in the sample. Nevertheless, a stepwise replication [30] of this study, but with data from pregnant women and partners who had chosen to continue the pregnancy after a CHD diagnosis [31] confirmed the stability of data over conditions.

Although persons with different cultural backgrounds were represented, a majority of the parents in this study had an educational level at or above senior high school. Furthermore, the sample lacks parents with Swedish as a second language in need of an interpreter service.

It is important to bear in mind that this study only sheds light on the experiences of those choosing to continue the pregnancy following a CHD. Those who terminate the pregnancy after a prenatal diagnosis of CHD may have different experiences regarding the information process.

Taken together, we consider the findings credible and worth taking into account when counseling pregnant couples in antenatal care who are faced with a fetal diagnosis. We consider it reasonable that the findings can be transferred to similar populations who have experienced a severe prenatal diagnosis. The transferability to settings including persons with low health literacy skills and language difficulties and to persons who choose to terminate the pregnancy needs, however, to be addressed in future studies.

### Clinical Implications and Suggestions for Future Research

This study generated hypotheses to be tested in future research, namely information provision and counseling, antenatal parental education and information provided via the Internet following a prenatal diagnosis of CHD.

Findings from this study highlight the importance of customizing the information given following a prenatal diagnosis of CHD so that the information is based on the parent’s individual preferences. However, further research is required to evaluate different approaches of information provision and counselling [32] with special attention directed towards non-native speakers and persons with low health literacy skills.

Parents to children prenatally diagnosed with CHD move from grief to preparation for the future as the pregnancy progresses [20]. Consequently, caregivers need to take responsibility for the provision of antenatal education to these parents. This and other studies [3,8,16] conclude that the teaching should be based on the parents’ needs for balanced, honest, and realistic information so that they know what to expect and later on will be able to make informed decisions. In addition, antenatal group education may encourage and develop lasting social support [33], which was called for by the parents in this study.

The parents wanted an easily accessible information source via the Internet to complement standard care after a prenatal diagnosis of CHD. The Internet [34] and other technology-based applications [35] have rapidly become a regular source of information among patients. Developing an easily accessible information source via the Internet that enables expectant parents to customize the information given based on individual circumstances may complement standard care. As a mean towards this end, national, or even international, collaborations is needed and the effectiveness of such tools need to be evaluated in future studies.

### Conclusions

Health professionals need to acknowledge several factors when providing information to expectant parents following a prenatal diagnosis of CHD. Firstly, early and honest information to meet individual preferences, with special attention to non-native speakers, is crucial to support the decisional process regarding whether to continue or terminate the pregnancy. Secondly, illustrations, as a complement to oral information, are a pedagogic tool that increases comprehension of complex medical information, as well as satisfaction with the information obtained. Thirdly, the overwhelming amount of information on the Internet calls for the development of an easily accessible and reliable information source via the Internet.
